# A New Deep Model for Detecting Multiple Moving Targets in Real Traffic Scenarios: Machine Vision-Based Vehicles

**DOI:** 10.3390/s22103742

**Published:** 2022-05-14

**Authors:** Xiaowei Xu, Hao Xiong, Liu Zhan, Grzegorz Królczyk, Rafal Stanislawski, Paolo Gardoni, Zhixiong Li

**Affiliations:** 1 School of Automobile and Traffic Engineering, Wuhan University of Science and Technology, Wuhan 430081, China; xuxiaowei@wust.edu.cn (X.X.); xionghao@wust.edu.cn (H.X.); zhanliu2021@wust.edu.cn (L.Z.); 2 Hubei Key Laboratory of Mechanical Transmission and Manufacturing Engineering, Wuhan 430081, China; 3 Department of Manufacturing Engineering and Automation Products, Opole University of Technology, 45758 Opole, Poland; g.krolczyk@po.opole.pl; 4 Department of Electrical, Control and Computer Engineering, Opole University of Technology, 45758 Opole, Poland; r.stanislawski@po.edu.pl; 5 Department of Civil and Environmental Engineering, University of Illinois at Urbana-Champaign, Champaign, IL 61820, USA; gardoni@illinois.edu; 6 Yonsei Frontier Lab, Yonsei University, Seoul 03722, Korea

**Keywords:** multiple target detection, improved YOLOv4, multi-scale detection

## Abstract

When performing multiple target detection, it is difficult to detect small and occluded targets in complex traffic scenes. To this end, an improved YOLOv4 detection method is proposed in this work. Firstly, the network structure of the original YOLOv4 is adjusted, and the 4× down-sampling feature map of the backbone network is introduced into the neck network of the YOLOv4 model to splice the feature map with 8× down-sampling to form a four-scale detection structure, which enhances the fusion of deep and shallow semantics information of the feature map to improve the detection accuracy of small targets. Then, the convolutional block attention module (CBAM) is added to the model neck network to enhance the learning ability for features in space and on channels. Lastly, the detection rate of the occluded target is improved by using the soft non-maximum suppression (Soft-NMS) algorithm based on the distance intersection over union (DIoU) to avoid deleting the bounding boxes. On the KITTI dataset, experimental evaluation is performed and the analysis results demonstrate that the proposed detection model can effectively improve the multiple target detection accuracy, and the mean average accuracy (mAP) of the improved YOLOv4 model reaches 81.23%, which is 3.18% higher than the original YOLOv4; and the computation speed of the proposed model reaches 47.32 FPS. Compared with existing popular detection models, the proposed model produces higher detection accuracy and computation speed.

## 1. Introduction

Multi-target detection in traffic scenes is critical for a driverless car, object tracking, and intelligent driver assistance [1]. There are many dynamic objects around vehicles in real traffic scenarios, e.g., vehicles, pedestrians, cyclists, etc. Commonly used multi-target detection algorithms often produce poor performance and a high missed detection rate when detecting traffic targets that are too small or occluded [2,3]. Therefore, fast and accurate detection of potentially dangerous targets around the vehicles is a key issue [4,5].

Traditional target detection algorithms mainly extract features through sliding windows, such as Histogram of Gradient (HOG) features and Deformable Parts Model (DPM) features [6]. For example, Rao et al. [7] performed pedestrian detection by extracting the HOG features from candidate regions. Sun et al. [8] realized nighttime vehicle detection through a deformable part model. The main computational effort of these methods is consumed in the area selection of the sliding window, resulting in low robustness in complex environments (e.g., partial occlusion, small targets, and poor visibility in bad weather).

With the rapid development of convolutional neural networks (CNN), deep learning-based target detection techniques are widely used in traffic target detection. Compared with traditional target detection techniques, deep learning-based methods can extract complex feature information and be applied to complex detection environments [9,10]. Currently, deep learning-based traffic target detection algorithms are mainly divided into two categories. The first category is a two-stage target detection, including the Faster Region-based CNN (R-CNN) [11], Mask R-CNN [12], and Cascade R-CNN [13]. This category usually uses the Region Proposal Network (RPN) to extract candidate frames and detect the traffic targets, producing high detection accuracy but slow computation speed. The other category is the regression-based one-stage target detection algorithms, including the SSD [14] and YOLO [15,16,17] algorithms. This category directly provides the location and class information of the target; the computation speed is fast, but the detection accuracy is usually lower than that of the first category.

Many researchers have applied these algorithms to target detection in intelligent driving. According to the complex factors of traffic targets, scholars have made corresponding improvements to the algorithm to improve the detection accuracy and speed of traffic targets. Han et al. [18] introduce a feature fusion network and an adversary occlusion network based on the Faster R-CNN structure, which increases the ability to extract low-level features in the network and improve the detection of small and occluded targets. Zhong et al. [19] integrated a bidirectional feature pyramid network (BiFPN) into Cascade R-CNN, which used the BiFPN structure to connect multiple scales to more effectively fuse weighted features, thereby enhancing the feature extraction ability of the network and improving the detection effect of occluded and small targets. The YOLOv3 algorithm introduces a feature pyramid network (FPN), which has achieved good speed and accuracy Performance. Ju et al. [20] used four detection layers based on YOLOv3 to enhance the performance of detecting small targets and reduce the missed detection rate of small targets. Cai et al. [21] designed 5-scales detection layers based on YOLOv4 to improve the detection accuracy of small targets. Guo et al. [22] improved the YOLOv3 algorithm by fusing features by adding a spatial pyramid pool and an attention mechanism, which improved the detection accuracy of vehicles and pedestrians. In summary, these efforts improve the detection of the algorithm by increasing the effective features of small targets with the help of feature fusion or adding scale detection layers.

Recently, the attention mechanism has been proven to enhance deep convolutional features and improve target detection performance [23,24]. Hu et al. [25] proposed the channel attention module, which can adaptively acquire the importance of each feature map using learning. In this way, the importance of useful features was increased while reducing the importance of useless features. The proposed channel attention modules were applied to the ResNet and ResNeXt series of networks and achieved good detection results on the ImageNet2012 dataset. In addition, Woo et al. [26] developed the Convolutional Block Attention Module (CBAM), which models the channel relations and spatial relations between convolution operations at the same time can better filter out the required features. CBAM also has wide applicability to other networks. Due to its intuitiveness and versatility. The attention mechanism has received extensive attention in the field of target detection and has shown great potential. Therefore, the introduction of the CBAM module in the neural network to weight the features of the target region. It can enable better localization over the features to be detected and also improve the generalization performance of the network while not introducing too many parametric quantities [27,28,29,30].

In intelligent driving perception systems, the target detection algorithms must make a good trade-off between detection speed and accuracy. Compared with the two-stage target detection, the one-stage methods can balance detection speed and accuracy. Therefore, an improved YOLOv4 method is proposed for multi-target detection of traffic scenes, in which detecting small and occluded targets are addressed. Firstly, to detect small targets, shallow feature map information is added to the YOLOv4 model, expanding the original three-scale detection structure to a four-scale detection structure; then, an attention mechanism is introduced in the YOLOv4 neck network to supplement the feature information for small and occluded targets; finally, in the prediction phase, the DIoU (distance-intersection over union) is introduced in the soft-NMS (non-maximum suppression) to improve the detection of occluded targets. Experimental evaluation demonstrates the effectiveness of the proposed method.

## 2. Materials and Methods

**Figure 1:**
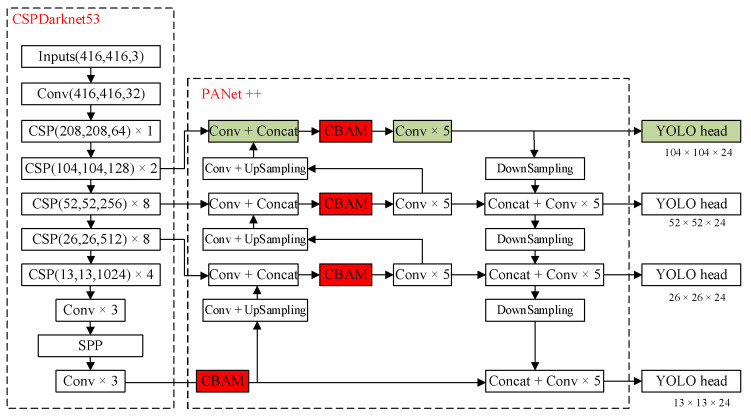
Improved YOLOv4 algorithm framework.

To improve the detection effect in a complex traffic environment, this paper improves the original YOLOv4 by (1) expanding the original 3-scale structure to a 4-scale structure, (2) adding CBAM into each scale, and (3) introducing the DIoU-based Soft-NMS to enhance the occluded target detection rate. The improved network structure is shown in Figure 1.

### 2.1. Four-Scale Detection

In actual driving scenes, there are a large number of small targets, such as pedestrians and distant vehicles, which have fewer pixel points and less obvious features in the image. The maximum detection scale of the original YOLOv4 is obtained by down-sampling the input image by a factor of 8, and the number of feature extraction layers is deep, which easily causes the loss of feature information of such small targets. As shown in Figure 1, a detection layer of size 104 × 104 is added to the 3-scale detection layer of the YOLOv4 network. The neck network structure is adjusted to perform a 2× up-sampling operation on the 52 × 52 size feature mapping in the pyramid network structure, which is overlaid with the 104 × 104 sized feature mapping generated by the backbone network to create a feature fusion target detection layer with 4× down-sampling as output; then the 104 × 104 size feature map is down-sampled by adding a new PAN structure, which concatenates with the 52 × 52 size feature map to form a new 8× down-sampling feature map, completing the bottom-up feature fusion operation of the neck network. Compared with other scale detection layers, the 104 × 104 size detection layer can obtain more refined feature information and improve the detection effect on small targets.

### 2.2. Introduction of CBAM

**Figure 2:**
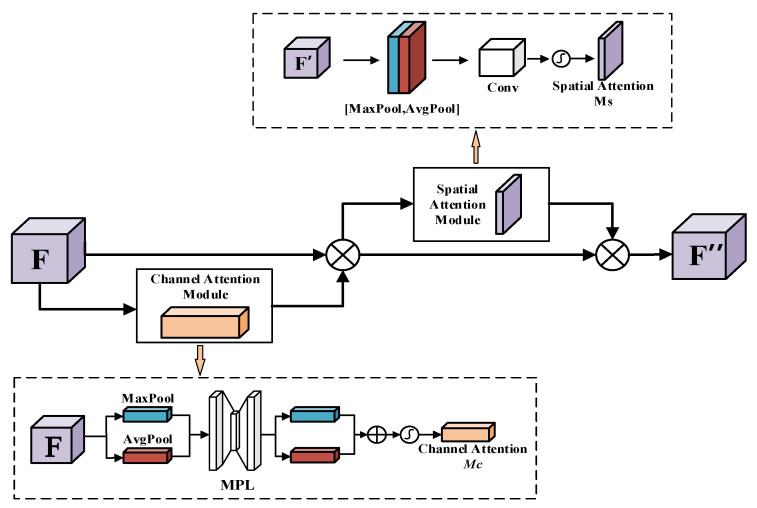
Architecture of CBAM. The module has two sequential sub-modules: channel and spatial.

The attention mechanism in deep learning refers to focusing on important information in an image and ignoring invalid information, as in human vision. The attention mechanism can weigh the weights of different feature channels, making the network focus more on the target region of interest and guiding the network to highlight useful features for the current network task [20]. Therefore, in this paper, a CBAM [21] is added to each of the four detection scales to enhance the model’s ability to learn feature information and improve the detection accuracy of the model. As shown in Figure 2, the CBAM consists of a channel attention module and a spatial attention module. The channel attention module focuses on different channels of the input feature map to enhance the weight of key features, and the spatial attention module enhances the localization of critical features on this basis. Its mathematical expression is described as:

(1) $$\begin{array}{c} F^{\prime }=M_{C}(F)⊗F\\ F^{\prime \prime }=M_{S}(F^{\prime })⊗F^{\prime } \end{array}$$

which ⊗ denotes the corresponding multiplication of pixel values, *F* represents the input feature map, *M_C_* represents the channel attention weight coefficients, *M_S_* represents the spatial attention weight coefficients, *F*^′^ is the feature map out by the channel attention module, and *F*^″^ represents the feature map output by the CBAM module.

In Figure 2, the channel attention module first performs global maximum pooling and average pooling operations on the feature map *F* to obtain the feature information of each channel firstly and then performs operations of dimension reduction and dimension enhancement on the Multi-Layer Perceptron (MPL), which composed of two fully connected layers to obtain two feature channel attention vectors, finally, the two vectors outputted by MPL are summed and pass through the Sigmoid nonlinear activation function to obtain the channel attention weight coefficients *M_C_*. The channel attention module re-weights each channel feature of the input feature map *F* by *M_C_* to increase the effective channel weights and suppress the invalid channel weights. The attention weight coefficient *M_C_* is described as:

(2) $$\begin{array}{c} M_{C}=\sigma \left(MLP\left(AvgPool\left(F\right)\right)+MLP\left(MaxPool\left(F\right)\right)\right)\\ =\sigma \left(W_{1}\left(W_{0}\left(F_{avg}^{c}\right)\right)+W_{1}\left(W_{0}\left(F_{\max}^{c}\right)\right)\right) \end{array}$$

where σ denotes the Sigmoid activation function, *W*_0_ and *W*_1_ denote the two fully connected layer weight matrices in the multiple layer perceptron, $F_{avg}^{c}$ and $F_{\max}^{c}$ respectively denote the out feature through average pooling and maximum pooling.

In Figure 2, the spatial attention module compresses the channel information using global maximum pooling and average pooling operations on the feature map *F′* obtained through the channel attention module, and splices the two-channel information together to obtain a two-channel feature, then which is reduced in dimension using a 7 × 7 convolution kernel, and finally the spatial attention weight coefficients *M_S_* are obtained after a Sigmoid activation function. *M_S_* is defined as

(3) $$M_{S}=\sigma (f^{7\times 7}([AvgPool(F);MaxPool(F)]))$$

where *σ* denotes the Sigmoid activation function, *f*^7 × 7^ represents convolution operation.

### 2.3. Soft-NMS

Most target detection algorithms use the NMS algorithm in the post-processing stage, where the NMS is used to filter the bounding boxes and only a portion of the bounding boxes are retained for the final target location. The traditional NMS algorithm forces the deletion of bounding boxes with low confidence if the Intersection Over Union (IoU) value of two bounding boxes is greater than the set overlap threshold, the bounding boxes with low confidence will be forcibly removed, which leads to easily missed detection of targets, especially in scenes with target occlusion. Therefore, we use the Soft-NMS algorithm [22] instead of the NMS algorithm. Unlike the traditional NMS algorithm, the Soft-NMS algorithm uses a weight decay function to suppress the confidence of the bounding box, the current bounding box confidence is multiplied by a weight function, this function decays the confidence of the bounding boxes, which overlaps with the highest confidence bounding box, to retain the low confidence of the occluded targets. In addition, DIoU [23] considers the center point distance and overlap area of the bounding box, which can better converge. Using DIoU instead of IoU to calculate the similarity between targets can more accurately portray the relative position and overlap between targets. Finally, a Gaussian penalty function is used to predict the box confidence decay coefficient. The mathematical formulation is described as

(4) $$S_{f}=\{\begin{array}{cc} S_{i}, & DIoU\left(M,b_{i}\right)<N_{t}\\ S_{i}e^{-\frac{DIoU{\left(M,b_{i}\right)}^{2}}{\sigma }}\; & ,DIoU\left(M,b_{i}\right)\geq N_{t} \end{array}$$

where: *b_i_* is the *i*-th bounding box to be predicted, *S_i_* is the original score of *b_i_*, *S_f_* is the final score of *b_i_*, *M* is the bounding box with the highest score, *DIoU* (*M, b_i_*) is the distance intersection over the union of *b_i_* and *M*, and *N_t_* represents the threshold for screening two overlapping boxes, which is set to 0.3 [22]. When the overlap between the predicted box *b_i_* and *M* is greater, the smaller the value of *S_f_* is, the stronger the suppression effect is. As a result, avoiding missed detection due to forced deletion of predicted boxes and improving target detection in occlusion situations.

## 3. Experiments and Results Analysis

The experiments using the KITTI and BDD100K datasets demonstrate that the improved YOLOv4 can improve detection accuracy and speed for traffic targets over related models. All experiments were trained and tested in the PyTorch framework, with the version of CUDA and cuDNN being 10.0 and 7.4, respectively, and an Nvidia RTX2080Ti graphics card as the hardware configuration.

### 3.1. Evaluation Indicators

To verify and evaluate the effectiveness of the improved YOLOv4 network in this paper, the commonly used Average Precision (AP), mean Average Precision (mAP), and detection speed (FPS) were selected as evaluation indicators. In the prediction, the IoU value between the target predicted bounding box and the real bounding box is used as an indicator of whether the sample is correctly predicted, and the threshold value of IoU is set to 0.5, i.e., samples with an IoU value greater than 0.5 are regarded as positive samples detected, otherwise they’re negative sample. AP is the average precision of the model for a certain kind of target detection, and mAP is the average precision of the model for all classes of detection judgments, and the calculation formula is described as (5).

(5) $$\{\begin{array}{c} AP=\int_{0}^{1}P(R)dR\\ mAP=\sum_{i=1}^{N}AP_{i}/N \end{array}$$

where, *N* is the number of all classes. Where the check-all rate *P* represents the ratio of the number of correctly predicted samples to the total number of samples, and the recall rate *R* is the ratio of the number of correctly predicted samples to the number of marked true samples. The check-all rate *P* and the recall rate *R* can be calculated as

(6) $$\{\begin{array}{c} P=TP/(TP+FP)\\ R=TP/(TP+FN) \end{array}$$

where True Positive (*TP*) represents samples whose predicted target class is consistent with the true target class; False Positive (*FP*) represents samples whose predicted target class is inconsistent with the true target class; False Negative (*FN*) represents samples whose true target exists but is not predicted by the network.

### 3.2. Experiment Based on KITTI Dataset

The KITTI object detection dataset, commonly used for computer algorithm evaluation in autonomous driving scenarios, and is based on real data collected from urban, rural, and highway scenarios and contains many complex scenarios, such as occluded vehicles, pedestrians, and cyclists. The KITTI dataset consists of 7481 images from the test set and 7518 images from the training set.

As the label information in the test set was not publicly available, the training set images are re-randomly divided into a new training set and a test set according to a ratio of 8:2, and the categories in the dataset were re-merged into three categories of objects, including car, pedestrian, and cyclist. The image size in the KITTI dataset was 1242 × 375, and for the purpose of algorithm effect comparison, the input images were re-scaled to a size of 416 × 416.

**Figure 3:**
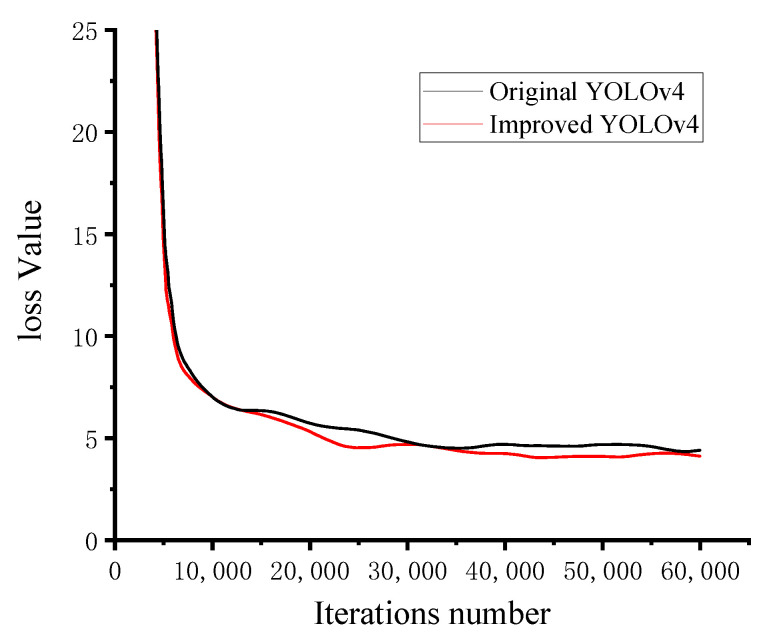
Graph of the change in training loss values.

The parameters were set as follows: the batch size was 8, the momentum was 0.9, the decay coefficient was 0.0005, the maximum number of iterations was set to 60,000, the initial learning rate was 0.001, and the learning rate changed to 0.0001 and 0.00001 at 15,000 and 35,000 iterations, respectively. In addition, to prevent overfitting of the model training, the training process was added to early stop. The variation of the loss value during training of YOLOv4 before and after improvement is shown in Figure 3, and the loss value region is stable after the number of iterations exceeds 30,000.

**Figure 4:**
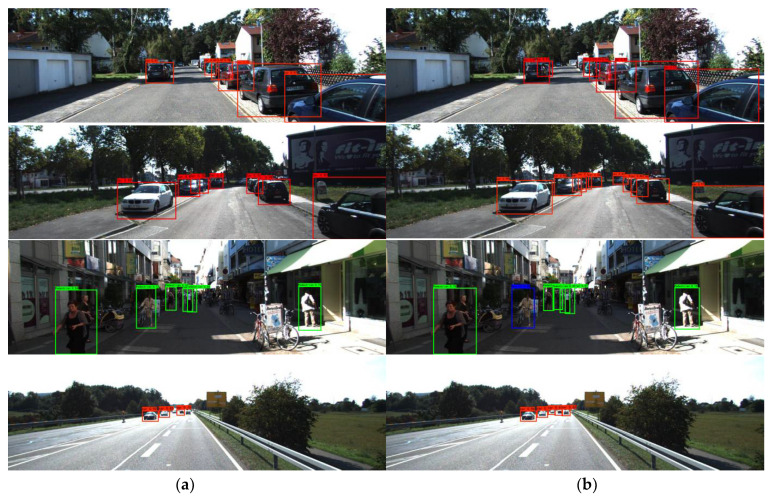
Comparison of detection results. (**a**) YOLOv4. (**b**) Improved YOLOv4. Red, blue, green box represent the labels of car, cyclist, pedestrian.

Figure 4 shows an example plot comparing the detection results of the YOLOv4 algorithm and the improved algorithm in this paper on the KITTI test sets, including a variety of complex scenarios. In the first and second row plots of Figure 4, the improved YOLOv4 algorithm detects better in a multiple vehicle occlusion, vehicle-dense environment, and the YOLOv4 algorithm misses some occluded vehicles. In the fourth figure of Figure 4, the improved YOLOv4 algorithm performs effective detection for small target vehicles at a distance, while the YOLOv4 algorithm produces more missed detection. From the example of the detection effect, it can be seen that the improved YOLOv4 algorithm in this paper accurately identifies obscured targets and smaller targets in complex traffic scenes and further improves the detection effect compared with the YOLOv4 algorithm.

To further verify that the proposed method can effectively improve the accuracy of traffic multi-target detection, the improved strategy proposed in this paper is compared with the original YOLOv4 algorithm on the KITTI dataset. The improved YOLOv4 traffic target detection algorithm consists of three improvements: adding a scale detection layer, introducing a hybrid attention module, and introducing a DIOU-based Soft-NMS algorithm. Each improved strategy is combined with the original YOLOv4 algorithm in turn, and the corresponding comparison experiments are conducted on the validation set to verify the effectiveness of each improved strategy. Experiment A used the original YOLOv4 algorithm for training, Experiment B added a scale detection layer to the model of the original YOLOv4 algorithm, Experiment C added the DIOU-based Soft-NMS algorithm to the model of Experiment B, Experiment D introduced a blending attention module to the model of Experiment C, and Experiment E added the DIOU-based Soft-NMS algorithm to the model of Experiment D. Table 1 shows the comparative experimental results of the four experimental models on the KITTI dataset.

Table 1 shows that in terms of the number of parameters, the improved YOLOv4 model parameters increased by 13.1 MB relative to the original YOLOv4 model parameters, introducing a smaller amount of additional computation and network computing, the speed of change is low; in terms of detection accuracy, the improved YOLOv4 model significantly improved the average detection accuracy by 3.18%. From the analysis of the changes in AP values of each model, model B with the addition of the scale detection layer, significantly improves the detection accuracy of small objects compared to the original YOLOv4 model, especially for the relatively small traffic targets of pedestrians and cyclists; model D introduces a CBAM on top of model B, and the model detection accuracy improves by 1.01%, which shows that the attention module has a great influence on the improvement of model detection accuracy; model C and model E introduce the DIOU-based Soft-NMS algorithm, and the model detection accuracy is further improved, and the detection accuracy is significantly higher than the other two categories in the pedestrian detection category, which proves the effectiveness of the DIOU-based Soft-NMS algorithm in dealing with the object occlusion problem in the traffic environment.

**Figure 5:**
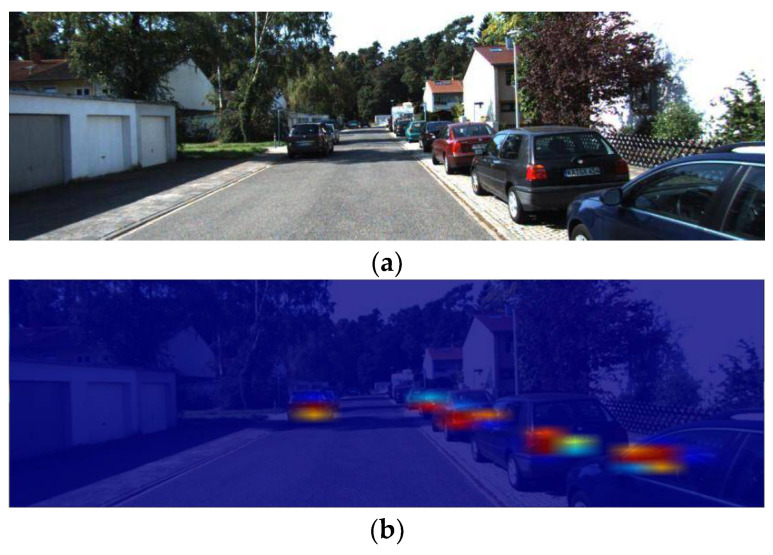

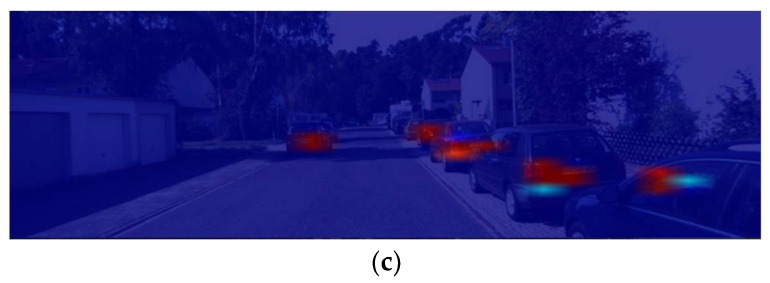
Visualized position prediction heat map with detection scale of 26 × 26. (**a**) Input image. (**b**) Heat map of yolov4 output. (**c**) Heat map of yolv4 + CBAM output.

To better visualize the impact of the CBAM module on the detector performance, a visualization of the location prediction activation plot is given in Figure 5. This also demonstrates that the attention module can direct the network to focus more on the visible part of the target while also reducing the impact of background noise on the detection performance.

In summary, all three types of improvement strategies are indispensable for improving the overall detection accuracy of the model. Compared with the original YOLOv4 model, the improved YOLOv4 model proposed in this paper can effectively detect multiple types of targets in traffic scenes while improving the detection accuracy when detecting small and occluded targets.

**Figure 6:**
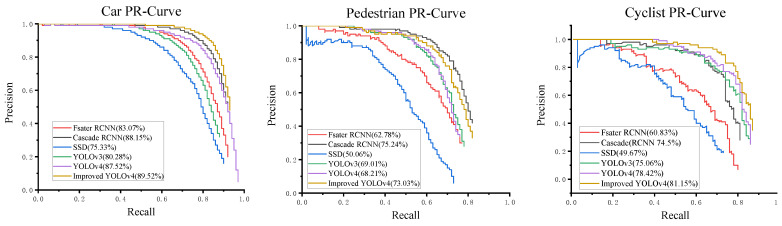
The precision-recall curves about different detection methods on the KITTI dataset.

To further validate the advancement of the improved YOLOv4 algorithm in this paper, compared with target detection algorithms such as Faster R-CNN, Cascade R-CNN, SSD, YOLOv3, and YOLOv4. Training and test evaluations were performed using the official code of each algorithm in KITTI test data, and the test results are shown in Table 2. PR curves of different detection methods on the test set are shown in Figure 6.

As can be seen from Table 2, compared with YOLOv4 with a detection speed of 51.68 FPS, the proposed algorithm in this paper improves the mean average precision (mAP) by 5.85% and can achieve real-time detection. The two-stage detection algorithms represented by Cascade R-CNN and Faster R-CNN have good mAP of 68.89% and 79.30%, respectively, in the KITTI test dataset, but the detection speed was too slow to meet the real-time requirements of autonomous driving perception systems. The single-stage detection algorithm SSD has a good detection speed in the test set, but the detection accuracy is too low. The YOLO series of detection algorithms have good performance in detection speed and accuracy, especially the YOLOv4 algorithm, which obtains 78.05% detection accuracy and 51.68 FPS detection speed. However, the improved YOLOv4 algorithm has less loss in detection speed and 3.18% higher detection accuracy than the YOLOv4 algorithm, while the detection speed reaches 47.32 fps, exceeding the YOLOv3 algorithm. The improved YOLOv4 algorithm has been shown to perform well in terms of speed and accuracy and meet the requirements of the autonomous driving scene detection algorithm.

### 3.3. Experiment Based on BDD100K Dataset

The BDD100k dataset is the largest open autonomous driving dataset with 100K videos and 10 tasks to evaluate the progress of image recognition algorithms on automatic driving. The dataset possesses geographic, environmental, and weather diversity, which is useful for training models that are less likely to be surprised by new conditions. It provided 100.000 images with a resolution of 1280 × 720 pixels. Due to the limited computing power of the device, we only used 10,000 images in BDD100K dataset for training and evaluation of the model, and the ratio of the training set to the testing set is 8:2. The training parameters of the model were set as in the above experiments. After data statistics and classification, the detection test results under different algorithms are illustrated in Table 3.

Table 3 demonstrates that the target detection accuracy of the improved YOLOv4 network model is higher than other detection algorithms under the BDD100K dataset. The two-stage detectors generally showed better detection accuracy compared to one-stage detectors, but the detection speed was too slow and not a balanced algorithm in terms of speed and accuracy. The SSD algorithm is not the best choice for speed and accuracy because the model runs detection for medium and small targets from very few layers. As a representative of one-stage detection algorithms, YOLOv4 has detected higher mAP values than other general-purpose target detection algorithms. It uses CSPDarkNet-53 as a backbone, which increases the accuracy of the classifier and detector. However, the improved YOLOv4 enhanced the mAP by 2.27% more than the YOLOv4 algorithm. For both pedestrian and bicyclist targets, the AP for each category of target identification increased by 3.4% and 1.75%; this indicates that our improved algorithm effectively detects small targets.

In summary, it can be seen that our improved YOLOv4 model not only outperforms other algorithms in terms of detection accuracy and recognition accuracy but also achieves a detection speed of about 46 frames/sec, which verifies that the algorithm in this paper can achieve a balance of accuracy and speed, with strong application prospects.

## 4. Conclusions

Multi-target detection algorithms must meet high detection accuracy and real-time detection speed in traffic scenes, especially high detection accuracy. However, generic multi-target detection algorithms have difficulty detecting small and occluded targets in complex environments. To address this problem, we propose an improved YOLOv4 algorithm, which is specifically designed for multi-target detection in traffic scenes. A new scale detection layer was added to the original YOLOv4 network to improve the algorithm’s ability to detect small targets, and the CBAM was introduced into the feature fusion network; finally, a Soft-NMS was used as a post-processing algorithm to improve the detection accuracy of occluded objects. Compared with the original YOLOv4 algorithm, the average accuracy obtained is improved by 3.18% on the KITTI dataset and 2.27% on the BDD100K dataset, which proves that the proposed improved strategy can effectively improve the detection accuracy of small and obscured targets. Compared with other multi-target detection algorithms, the proposed algorithm achieved an average accuracy of 81.23% for three types of detection targets while maintaining a detection speed of 47.32 FPS, which is both a good detection speed and detection accuracy and can complete the task of multi-target detection in traffic scenes very well. The method proposed in this paper has good application scenarios in various traffic scenarios, but in the face of harsh traffic environments, such as rain and fog, the method in this paper needs to be further improved.

## Figures and Tables

**Table 1:** Experimental results comparing before and after the improvement of the YOLOv4 algorithm.

Models	Improvements	AP@0.5 (%)			mAP@0.5 (%)	Model Size (MB)
		Car	Pedestrian	Cyclist		
A	YOLOv4	87.52	68.21	78.42	78.05	256.2
B	A + Add scale detection layer	88.31	71.06	80.45	79.94	258.7
C	B + DIoU-based Soft-NMS	88.53	71.31	80.54	80.13	258.7
D	B + CBAM	89.15	72.68	81.02	80.95	269.3
E	D + DIoU-based Soft-NMS	89.52	73.03	81.15	81.23	269.3

**Table 2:** Experimental results compared with other algorithms on the KITTI dataset.

Algorithms	AP@0.5 (%)			mAP (%)	FPS (Frames/s)
	Car	Pedestrian	Cyclist		
Faster R-CNN	83.07	62.78	60.83	68.89	14.21
Cascade R-CNN	88.15	75.24	74.50	79.30	8.20
SSD	75.33	50.06	49.67	58.35	45.13
YOLOv3	80.28	69.01	75.06	74.78	40.93
YOLOv4	87.52	68.21	78.42	78.05	51.68
Improved YOLOv4	89.52	73.03	81.15	81.23	47.32

**Table 3:** Experimental results compared with other algorithms on the BDD100K dataset.

Algorithms	AP@0.5 (%)			mAP (%)	FPS (Frames/s)
	Car	Pedestrian	Cyclist		
Faster R-CNN	60.02	48.83	46.17	51.67	13.10
Cascade R-CNN	65.77	50.41	47.36	54.51	7.40
SSD	50.35	39.26	38.76	42.79	44.52
YOLOv3	62.72	47.60	48.32	52.88	40.28
YOLOv4	72.26	50.86	54.78	59.30	51.45
Improved YOLOv4	73.92	54.26	56.53	61.57	46.83

## Data Availability

All data can be requested from the corresponding author.
